# Functional gut microbiomes enhance performance in house fly larvae

**DOI:** 10.1128/aem.00011-26

**Published:** 2026-04-13

**Authors:** Asmus Toftkær Muurmann, Jacob Agerbo Rasmussen, Morten Tønsberg Limborg, M. Thomas P. Gilbert, Simon Bahrndorff

**Affiliations:** 1 Department of Chemistry and Bioscience, Aalborg University 348577, Aalborg, Denmark; 2 Center for Evolutionary Hologenomics, University of Copenhagen 4321, København, Denmark; 3 Cmbio, København, Denmark; 4 University Museum, NTNU 8018, Trondheim, Norway; Norwegian University of Life Sciences, Ås, Norway

**Keywords:** house fly, metagenomics, host-microbe interactions, by-products, insects, side-streams

## Abstract

In a world with an increase in human population, food consumption, and the generation of organic waste, insects are emerging as a promising tool to convert organic waste material into human food or animal feed. The insect microbiome is known to play a key role in the degradation of organic substrates, but little is known about the metabolic potential of the microbiome of industrially reared fly larvae. We investigated the microbial composition and metabolic potential of the house fly (*Musca domestica*) larva gut microbiome from larvae grown on three different waste and by-product-based substrates. We found that bacteria associated with the larval gut were enriched for functions related to microbial stress mechanisms, indicating strong selection of the gut microbiome by house fly larvae. In addition, the gut microbiome of larvae reared on sludge-based substrate had higher diversity when weighting for rare species and a higher coverage of “carbohydrate transport and metabolism” genes compared to brewery by-product-based substrate. A positive correlation between coverage of “pyridoxal-P synthesis” and larval survival and substrate conversion efficiency suggests that microbial synthesis of vitamin B_6_ could enhance larval performance. Additionally, a negative correlation between coverage of the “Entner-Doudoroff pathway” and “homoprotocatechuate degradation” and substrate conversion indicates microbial competition for sugars and aromatic amino acids. Together, these results reveal how the host selects on gut microbiomes with metabolic potential that is optimized toward the conversion of substrates that may be ultimately valuable for commercial insect production.

**IMPORTANCE:**

Fly larvae are expected to play an important role in future food and feed production through the conversion of low-value biomass into high-quality protein. The gut microorganisms of fly larvae are expected to play an important role in bioconversion and could potentially be manipulated to improve biomass conversion. In this study, the importance of the gut bacteria of house fly larvae for bioconversion was investigated by metagenomic sequencing, which provided information on the bacterial abundance and potential functional roles in the larval gut. The results reveal that the functional potential of gut bacteria is affected by larval feed and correlates with larval performance, highlighting the importance of the gut microbiome for efficient biomass conversion.

## INTRODUCTION

The global human population is increasing, and together with an increasing urbanization and income growth in developing countries, there is an increasing demand for food and feed and for animal source proteins in particular (1, 2). Complicating this is the expanding volume of organic wastes that originate from the food and feed production line (3). Together, this not only presents challenges to global food security but also the environment with land use changes, eutrophication, and emission of greenhouse gases (4–6).

Insects have emerged as an attractive future food compared to conventional animal-based foods but also as feed (7–9). Insects are recognized for their nutritional and environmental advantages (10), and much of this has to do with their ability to effectively convert a range of organic substances, including low-degradable waste and by-products, into insect biomass. The high feed conversion efficiency of insects and remarkable ability to utilize waste and by-products from food production, including low degradable by-products, is largely due to the co-evolution with their gut microbiome communities (11, 12). Symbiotic interactions of insects with microbes are known to play important roles in digestion, detoxification, synthesis of essential nutrients, and immune stimulation and development (11, 13, 14). Less is known about the microbiome of insects used in commercial insect production, even though their microbiomes have a huge potential to convert organic waste and by-products into food and feed. We know that contributions of bacteria can contribute significantly to the degradation of sub-optimal diets (15–18), but we have an incomplete understanding of the importance of host-microbiome interactions critical for being able to feed different organic waste and by-products to insects. If we are to better utilize and valorize different organic waste and by-products in insect production, we need an improved understanding of the host-microbiome interactions, and the metabolic potential and importance of the microbiome in commercial insect production (19). In doing so, we can help improve production through, for example, increased flexibility in the use of diets, more stable production, and increased larval size. For example, the performance of insects used in commercial insect production often varies much across various substrates, making it necessary to deal with this instability in industrial production (20–23). By utilizing microbes as additives to insect feed, it has been shown that insect performance can be improved (24–28). This suggests an efficient way to stabilize insect performance to a variety of rearing substrates by adopting probiotics tailored for specific substrates. One challenge of probiotics, however, is overcoming barriers in probiotic establishment in the larval gut (29), although the chance of successful establishment can be improved by leveraging on microbes that are found natively in the host (30, 31).

In this study, we analyze differences in the house fly (*Musca domestica*) larva gut microbiome, as well as the substrate microbiome, across three waste and by-product-based substrates (a brewer’s spent grain-based substrate, a digested sludge-based substrate, and a wheat bran/deproteinized grass-based substrate), to reveal microbial adaptation to both different substrates and to the larval gut. When grown on these different substrates, both larval growth and metabolic performance traits are impacted, where the brewer’s spent grain-based substrate generally gave the highest performance in terms of larval weight, larval yield, and substrate conversion efficiency (32). To investigate the microbiome, we utilized long- and short-read sequencing to assemble and bin microbial reads into metagenome-assembled genomes (MAGs), allowing us to estimate the metabolic potential of the gut and substrate microbiome, in addition to taxonomy (33). Both larval gut and substrate samples were obtained in the study by Muurmann et al. (32) and allowed us to directly relate changes in larval microbiome with larval performance and to gain insight into metabolic pathways that correlate with different substrates and with the adaptation to the larval gut—information that is often lost when using only taxonomy as distinct taxonomic groups can perform similar metabolic functions (34). In addition, we analyzed correlations between larval phenotypic traits and coverage of microbial metabolic pathways. In doing so, we increase our understanding of larval gut microbial functions and their proliferation in the gut and on different waste and by-products. Both sources of information will be helpful for improving our understanding of the importance of host-microbiome interactions in commercial insect production.

## RESULTS

### Data overview from sequencing

**Fig 1:**
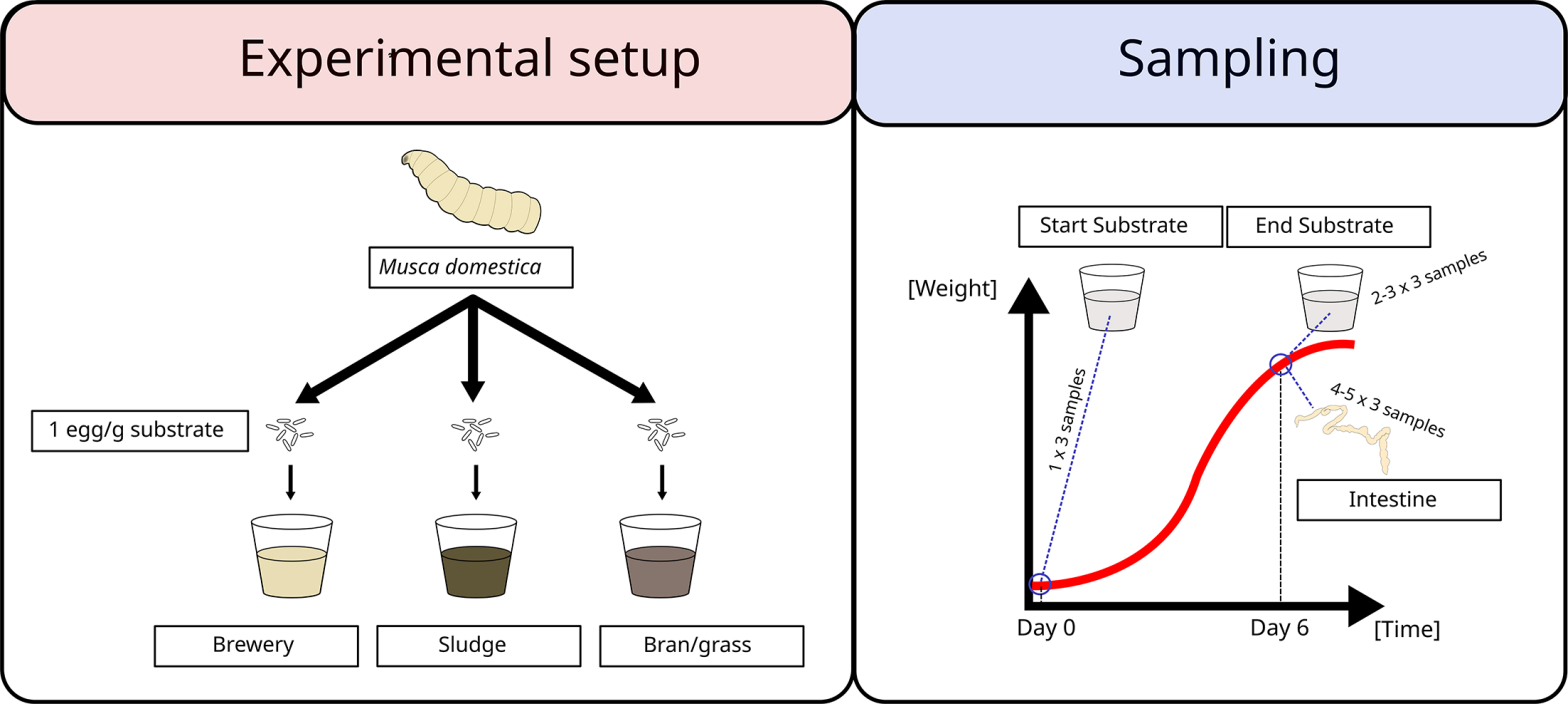
Overview of the experimental setup and sampling during the experiment. For the experiment, larvae of the house fly (Musca domestica) were grown on three substrates (brewery, sludge, and bran/grass), and substrate samples were sampled on days 0 and 6 (1–3 replicates per substrate), and guts on day 6 (4–5 replicates per substrate).

We reared house fly larvae on three different substrates and sampled gut and substrate samples for sequencing the microbiomes (see Fig. 1). Our microbiome analysis comprises both long-read and short-read sequencing, resulting in 76.2 Gbases and 166.7 Gbases, respectively (read quality metrics summarized in Data S2). Filtered reads were used for a hybrid assembly, where a co-assembly for each substrate was conducted, resulting in a total of 108,888 contigs generated, including 14,812 contigs for the brewery medium, 18,973 contigs for the bran/grass medium, and 75,103 contigs for the sludge medium. Contigs were made into scaffolds with a soft-split length of 20,000 bases for metagenomic binning (anvi’o default), resulting in 450,750 scaffolds comprising over 1.58 Gbases. Metagenomic-assembled genomes were generated using Metabat2 and further refined using anvi’o, resulting in a MAG catalog of 154 MAGs of at least 50% completion and one bin of unbinned contigs from 24 samples, including substrate and fly intestinal samples (see Data S3). In total, 147 MAGs met the medium-quality draft genome criteria (35), while 7 exhibited above 10% redundancy and therefore were of lower quality than a low-quality draft genome (see Data S3). Of the medium-quality MAGs, 8 lacked the 5S rRNA gene that is needed to be classified as high quality, while 35 additionally lacked the 23S rRNA gene, the 16S rRNA gene, or both. Taxonomic assignment of the MAGs in the catalog revealed it was comprised of two archaea, 136 bacteria, and 16 eukaryotes. Of the bacteria, the major phyla were Pseudomonadota (34.5%), Actinomycetota (33.8%), Bacillota (17.6%), and Bacteroidota (11.0%). Phylogenetic analysis showed that most bacterial MAGs could be taxonomically assigned at the genus level. However, a monophyletic cluster of nine MAGs that was most closely related to a MAG classified as *Pantoea* was not assigned any taxonomy (see Fig. S1). Some clusters had a phylogenetic distance of zero, indicating that these MAGs constitute the same species, likely an artifact from the MAG library generation, where MAGs were independently generated for each of the three substrates. Subsequent mapping of shotgun sequenced reads to the MAG catalog resulted in a total of 1.996 million mapped reads, constituting 1.2% of the total reads. The fraction of host reads was not tested, but earlier experiments with similar methodology suggest this number to be around 10% (unpublished data). Rarefaction curves for all samples were generated to investigate sequencing depth, which indicated sufficient sequencing depth, except for start substrate samples (see Fig. S2).

### Microbial dynamics in the intestine of *Musca domestica* is affected by substrate

**Fig 2:**
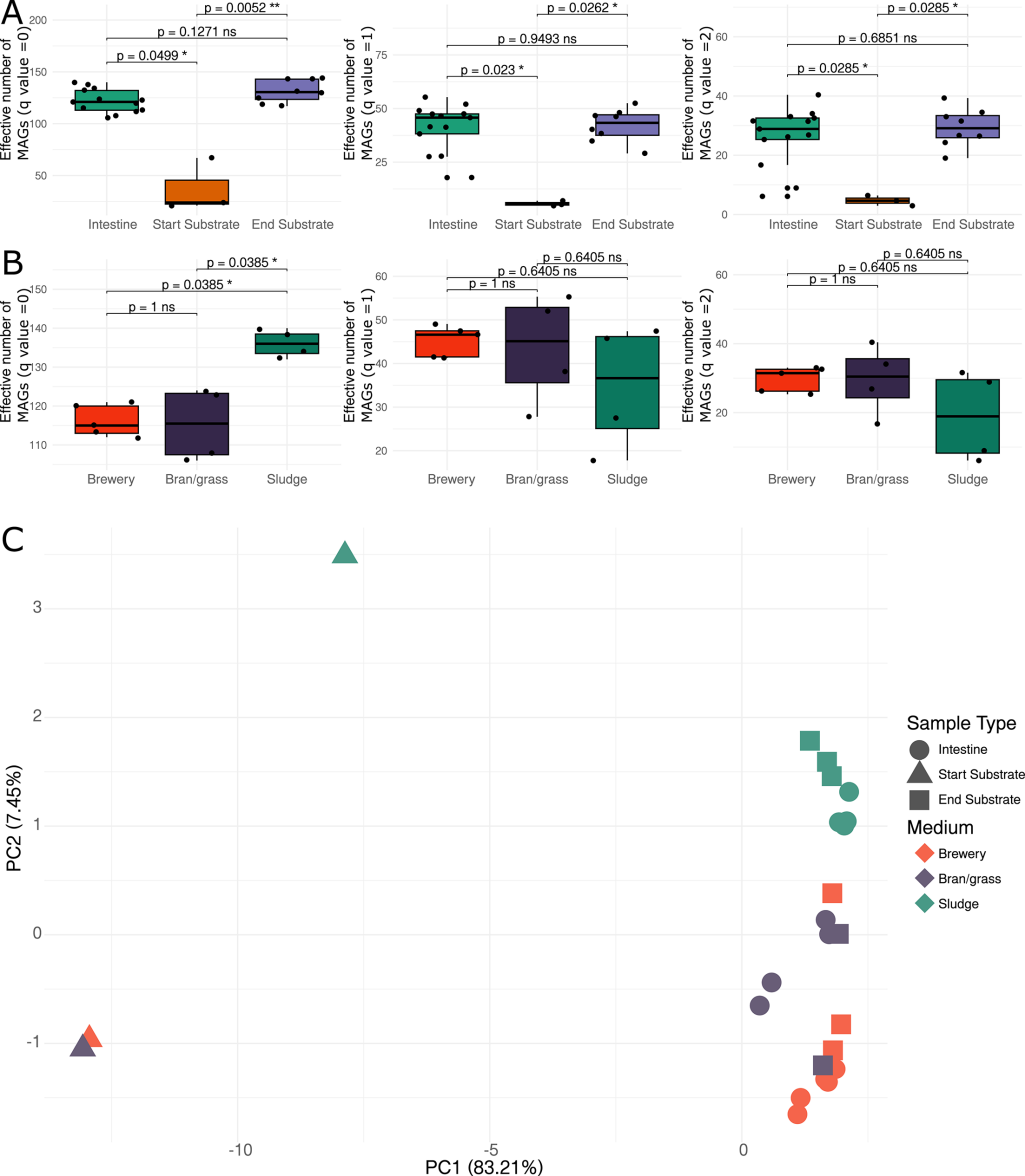
Metagenome dynamics related to substrate and sample types. (**A**) Boxplots of MAG diversity, using Hill effective numbers of MAGs across sample types, where *q* = 0 is MAG richness, *q* = 1 is Shannon diversity, and *q* = 2 is Simpson diversity. (**B**) Boxplots of intestinal MAG diversity, using Hill effective numbers of MAGs across substrates. (**C**) Principal coordinate analysis (PCoA) of metagenomes, based on the presence/absence of MAGs, related to sample types and substrate. The shape indicates the different sample types, and coloring indicates the different substrates.

Hill number-based analyses were used to characterize how the diversity of MAGs varied across samples. In the substrates, MAG richness (Hill number *q* value= 0) increased from day 0 to day 6 (see Fig. 2A). Furthermore, we saw a high richness in the intestinal microbiomes in the fly larvae compared to the starting substrate (day 0), but similar richness to the end substrate (day 6) (see Fig. 2A). Richness analysis across substrate revealed similar levels of the effective number of MAGs in intestines of larvae fed on bran/grass medium and brewery medium, while the highest richness was observed for the sludge medium (see Fig. 2B). When accounting for the abundance of MAGs (Hill number *q* value = 1 [Shannon diversity] and Hill number *q* value= 2 [Simpson diversity]), we saw no differences in diversity between fly larvae fed on different substrates. We noticed that the sludge medium resulted in the lowest effective number of MAGs (*q*-value = 1 and *q*-value = 2) in the intestinal samples, showing that the high number of observed MAGs in the fly larvae fed on the sludge medium is a result of low-abundant MAGs (see Fig. 2B).

Our investigation of the MAG profiles across samples revealed distinct microbiomes, related to both sample type and substrate. Ordination, using Principal Coordinates Analysis (PCoA), revealed clustering according to PC1 related to sample type (explaining 83.21%), and PC2 related to substrate (explaining 7.45%), where brewery medium and bran/grass medium were clustered together, and sludge medium clustered alone. Comparing binary and abundance-based ordination revealed separation of substrate, and sample type was defined by a shift of different MAGs (see Fig. 2C), as abundance-based PCoA did not show the same clustering as the clustering based on prevalence (see Fig. S3).

**Fig 3:**
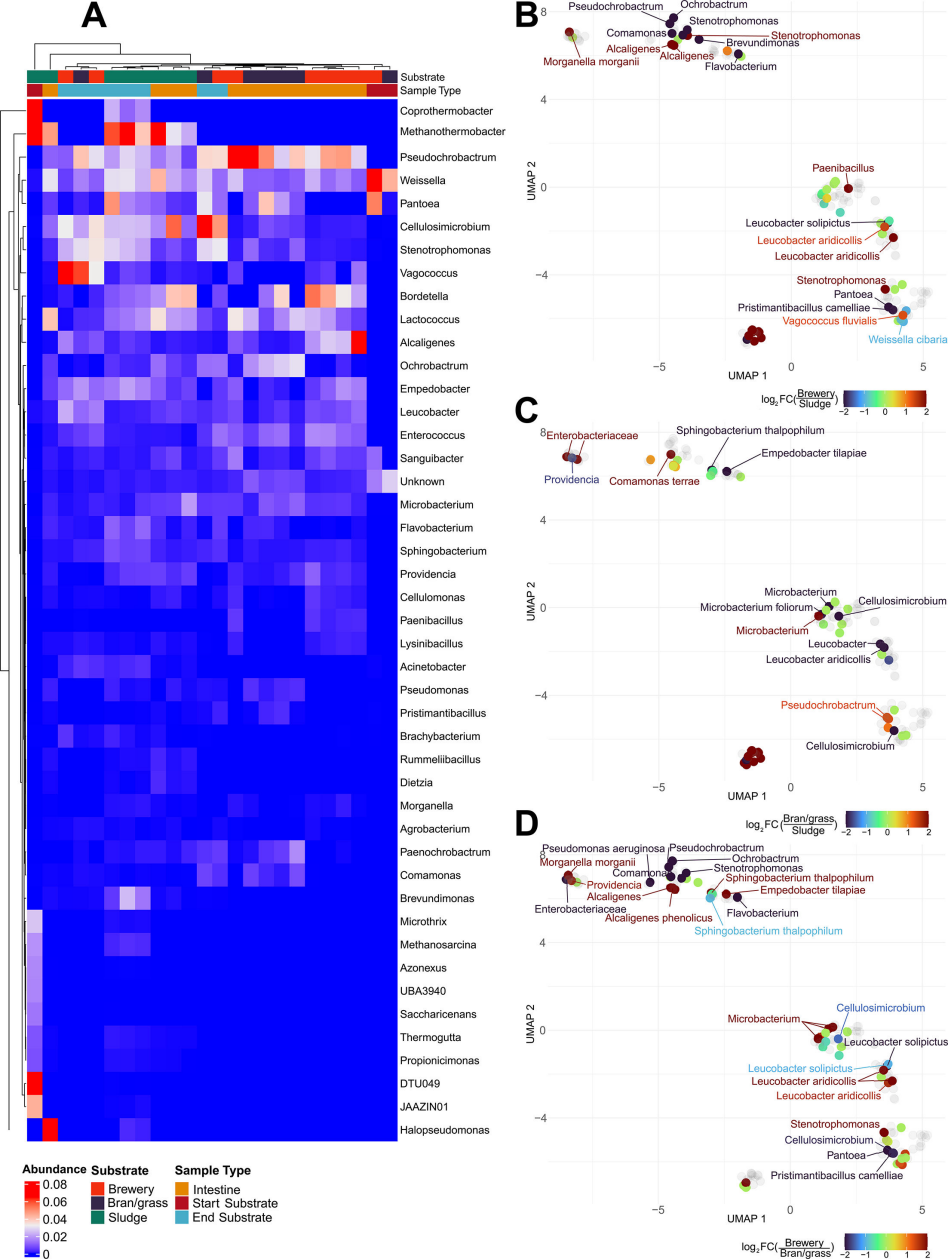
Metagenomic taxonomy and functional capacity across substrates. (**A**) Heatmap of summed normalized abundance of MAG genera across samples. Substrate and sample type are represented by color at the top panels. (**B–D**) Uniform Manifold Approximation and Projection (UMAP) of reconstructed metabolism of all MAGs. (**B**) Normalized abundance comparison of MAGs between brewery medium and sludge medium. (**C**) Normalized abundance comparison of MAGs between bran/grass medium and sludge medium. (**D**) Normalized abundance comparison of MAGs between brewery medium and bran/grass medium.

We constructed a heatmap based on the summed abundance of MAG genera across samples (see Fig. 3A) (see Data S4). The heatmap shows high abundance of the genera *Pseudochrobactrum* and *Weissella* across all substrate and intestine samples on day 6, and high abundance of the genera *Bordetella*, *Lactococcus*, *Microbacterium,* and *Leucobacter* in intestinal samples across all substrates. Specifically for the larvae reared on brewery medium, bacteria including *Alcaligenes*, *Empedobacter*, *Sanguibacter*, *Enterococcus*, *Paenibacillus,* and *Cellulomonas* were highly abundant in the intestine (see Fig. 3A). For the larvae reared on sludge medium, bacteria including *Cellulosimicrobium*, *Empedobacte*r, *Methanothermobacter,* and *Stenotrophomonas* were highly abundant in the intestine (see Fig. 3A). For the larvae reared on bran/grass medium, bacteria, including *Ochrobacterium*, *Paenochrobactrum*, *Pantoea*, *Stenotrophomonas,* and *Comamonas,* were highly abundant in the intestine (see Fig. 3A). In accordance with the Hill diversity metrics, only a few genera were detected in the start substrates while the diversity was higher in both intestine and substrate samples from day 6. Genera highly abundant in the substrate but not the intestine include *Coprothermobacter*, *Vagococcus*, *Brevundimonas*, *Acinetobacter*, and *Methanosarcina* (see Fig. 3A), indicating that these genera are substrate specific. Similar to the PCoA (see Fig. 2C), one of the substrate samples labeled as bran/grass medium clustered together with two of the brewery medium samples (see Fig. 3A), indicating that this first sample might be mislabeled.

We conducted a prevalence test to investigate which MAGs were related to the gut environment of the fly larvae. Using a prevalence threshold requiring 75% presence across intestinal samples coupled to no presence in the starting substrate, we identified 68 MAGs related to the fly gut (see Data S5). These MAGs were comprised of 13 bacterial genera, including *Microbacterium* (*n* = 15 MAGs), *Leucobacter* (*n* = 8 MAGs), *Enterococcus* (*n* = 4 MAGs), *Sphingobacterium* (*n* = 4 MAGs), *Alcaligenes* (*n* = 4 MAGs), *Lactococcus* (*n* = 3 MAGs), *Providencia* (*n* = 3 MAGs), and *Stenotrophomonas* (*n* = 3 MAGs). Furthermore, although the bacterial genus *Weisella* was found in all intestinal samples, it was also identified in the start substrate and therefore hypothesized to be more of a generalist than the other prevalent MAGs.

We used KOfams to predict the metabolic traits of the MAGs and to then compare how the metabolic potential of the MAGs varies between the gut communities associated with the three growth substrates (see Fig. 3B through D). This was done based on a UMAP projection of the KEGG module completion of each assembled MAG, and coloring of each MAG according to the log-2 difference in mean MAG abundance between two substrates. The UMAP projection reveals shifts in MAGs that potentially fulfill similar metabolic functions related to the substrates. In the far-left corner of Fig. 3B through D, there is a close clustering between MAGs of *Morganella morganii*, *Enterococcus*, *Providencia*, *Pseudomonas aeruginosa,* and *Enterobacteriaceae,* indicating that these MAGs have similar overall metabolic potential, although occurring at significantly different relative abundances between substrates. *Morganelli morganii* had higher abundance in larvae reared on brewery medium, *Enterobacteriaceae* had higher abundance in larvae reared on bran/grass medium, and *Providencia* lower, indicating that these different bacteria fulfill similar metabolic niches between substrates. Other clusters show similar shifts in microbial abundance of metabolically similar MAGs, with the top middle cluster showing *Alcaligenes* being more abundant in brewery medium, while *Pseudochrobactrum* and one MAG of *Stenotrophomonas* were less abundant. Another strain of *Stenotrophomonas,* that is located in another cluster (lower right cluster) had higher abundance in brewery medium, whereas *Pantoea* and *Pristimantibacillus camelliae* were less abundant compared to sludge medium and bran/grass medium. Several MAGs of *Leucobacter* were detected, forming a cluster in the right side of the UMAP projection, with great diversity in abundance differences between the various substrates. Finally, *eukaryotes* formed their own functional cluster at the middle bottom and were more abundant in brewery medium and bran/grass medium than sludge medium.

### Enrichment analysis reveals pathways associated with gut-related microbiome

**Fig 4:**
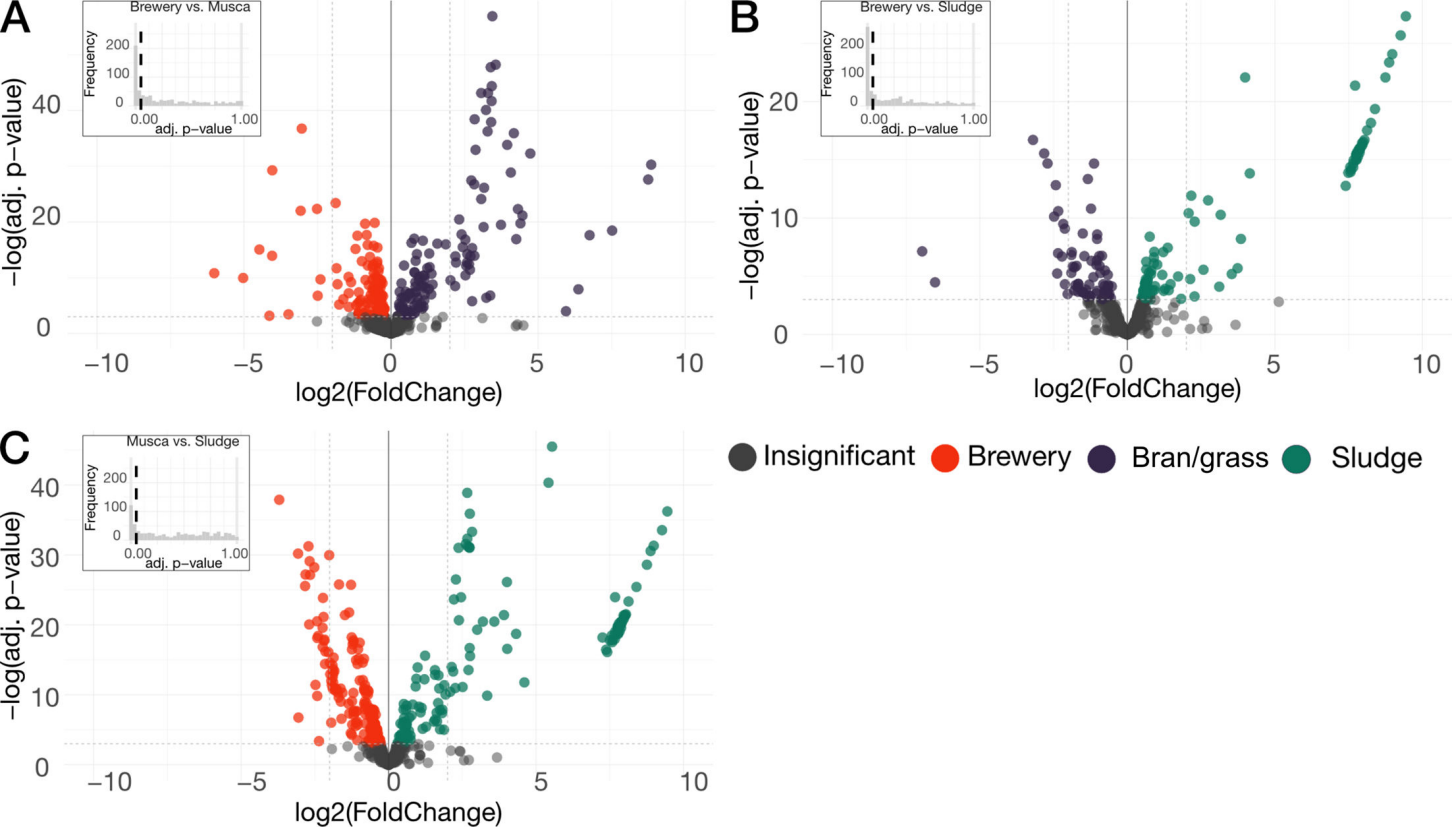
Enrichment analysis of KEGG modules of gut and non-gut-related MAGs. Volcano plot of enriched modules in gut- and non-gut-related MAGs. The X-axis indicates the enrichment score, and the y-axis indicates the adjusted *q*-value. Colors indicate the significance of modules, as indicated by the legend. (**A**) Comparison of brewery and bran/grass medium. (**B**) Comparison of bran/grass and sludge medium. (**C**) Comparison of brewery and sludge medium.

We investigated the metabolic potential of gut-related MAGs using an enrichment analysis that compared coverage across 280 microbial KEGG pathways (modules) for gut-related vs. non-gut-related samples (see Data S5). This revealed 31 (adjusted *q*-value < 0.07) modules that were highly related to gut-related MAGs, although only two of these modules were found significantly related to gut-related MAGs (adjusted *q*-value < 0.05). On the other hand, we only found two modules not related to gut-related MAGs (adjusted *q*-value < 0.05) (see Fig. 4; Data S6). Of the modules related to gut-related MAGs, we found enriched modules encoding for the functions “phenylacetate degradation” and “multidrug resistance.” Other non-significant modules of interest included “trehalose biosynthesis,” “phenylalanine biosynthesis,” “GABA (gamma amino butyrate) biosynthesis,” “GABA shunt,” “glycogen biosynthesis,” “urea cycle,” and “glyoxylate cycle.”

### Functional analysis reveals a shift in microbial dynamics related to substrate

We carried out a differential abundance analysis of the microbial functions based on read-depth-normalized summed coverage of the cluster of orthologous genes (COGs). When comparing brewery medium and bran/grass medium, we found 264 significantly differential abundant functions, where 134 COGs were found to be more abundant in intestines reared on brewery, and 130 COGs were found to be more abundant in intestines reared on bran/grass (see Dats S7). A comparison of the data from the brewery medium and sludge medium revealed that these groups were the most divergent, with 325 significantly differential abundant functions. Of the 325 COGs, 193 COGs were found to be more abundant in intestines reared on brewery medium, whereas in sludge medium, 132 COGs were found to be more abundant in intestines reared on sludge medium (see Dats S7). Lastly, when comparing bran/grass medium and sludge medium, we found 175 significantly differential abundant functions where 99 COGs were found to be more abundant in intestines reared on the sludge medium and 76 COGs were found to be more abundant in intestines reared on the bran/grass medium (see Dats S7).

**Fig 5:**
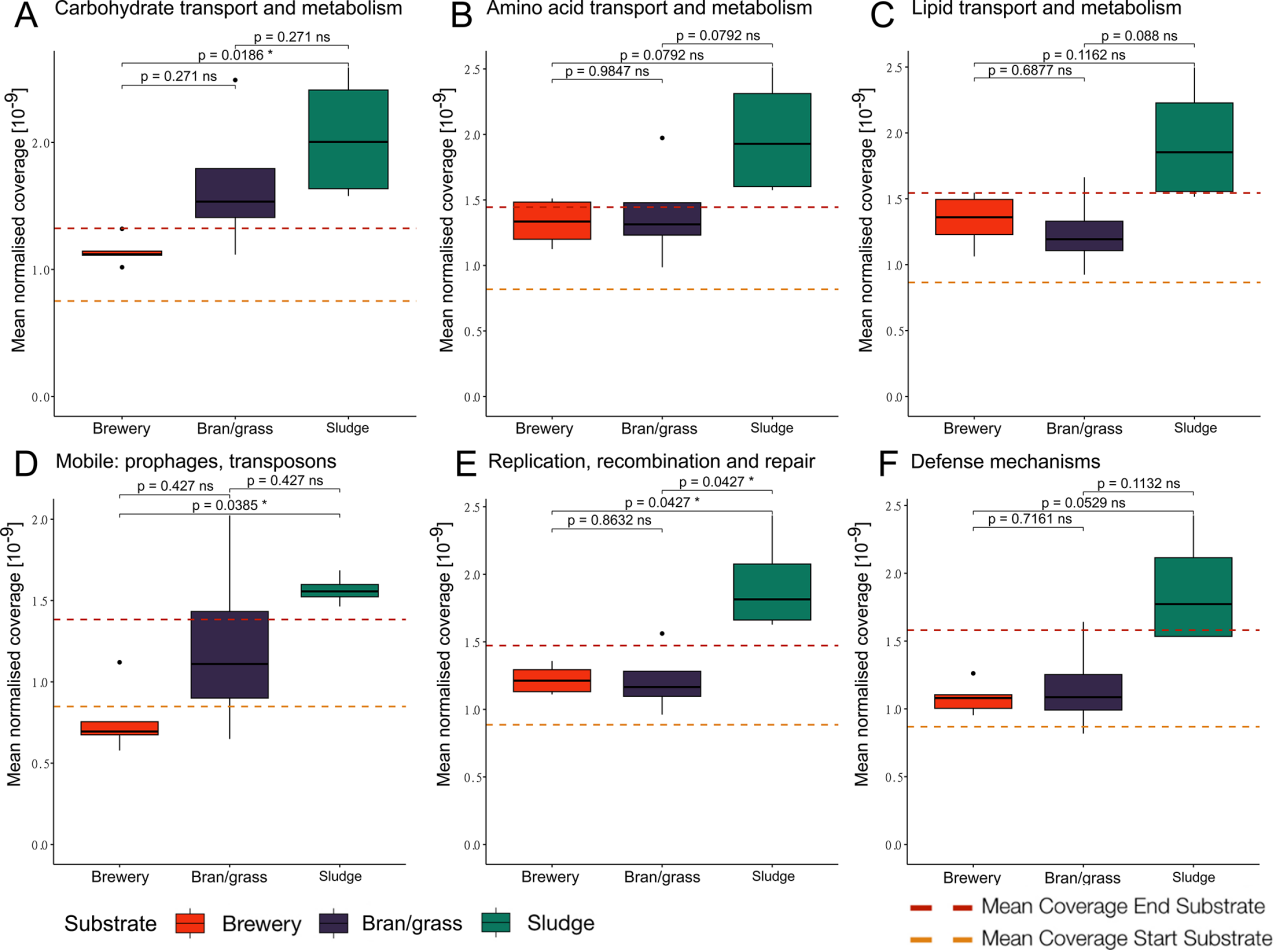
Pairwise comparison of mean abundance across substrates for modules of interest. (**A**) Carbohydrate transport and metabolism, (**B**) amino acid transport and metabolism, (**C**) lipid transport and metabolism, (**D**) mobile: prophage, transposons, (**E**) replication, recombination, and repair, and (**F**) defense mechanisms. The color legend indicates substrates. Dashed lines indicate the mean coverage of the module of interest across all substrates for both the start substrate (yellow) and the end substrate (dark red).

**Fig 6:**
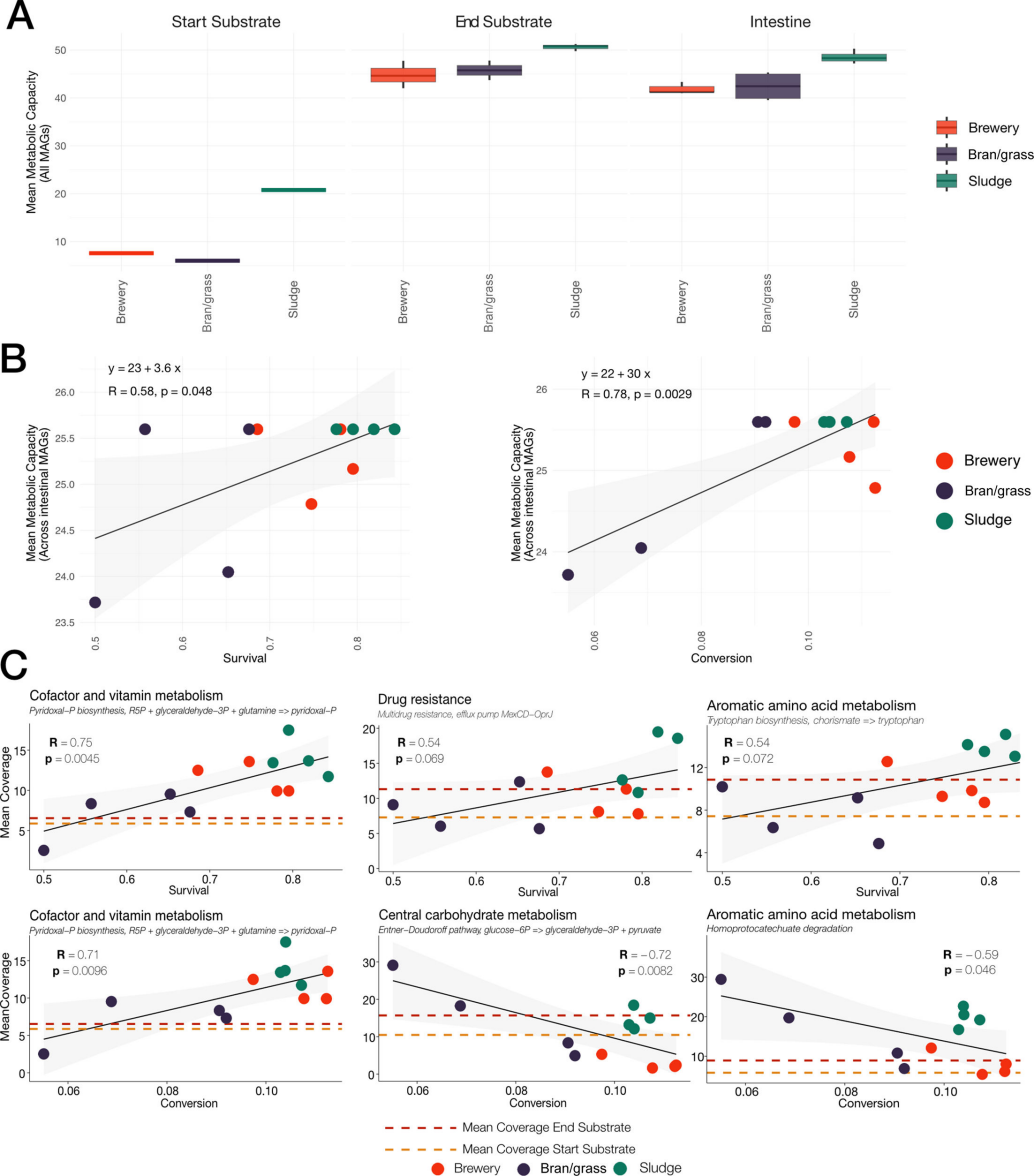
Correlation analysis of microbial functions, feed conversion, and survival of fly larvae. (**A**) Boxplots of metabolic capacity across all sample types and substrates. (**B**) Scatterplot of mean metabolic capacity for each sample of all substrates correlated with survival (left) and conversion of feed (right). The diagonal lines indicate a linear regression. The pale gray area indicates the confidence intervals of the regression. (**C**) Correlation analysis of selected KEGG module abundances and survival (top) and conversion (bottom). The diagonal lines indicate a linear regression. The pale gray area indicates the confidence intervals of the regression. Dashed lines indicate the mean coverage of the module of interest across all substrates for both the start substrate (yellow) and the end substrate (dark red). The color legend indicates substrates for all plots.

When exploring the metabolic categories using COG, we found that for most categories, intestinal samples of fly larvae reared on sludge medium had a higher normalized coverage in comparison to the start and end substrate, as well as intestinal samples from the other substrates, although rarely at a significantly higher level (see Fig. 5A through F). For the COG category “carbohydrate transport and metabolism,” there was a significantly higher mean coverage for samples from larvae reared on sludge medium than on brewery medium (see Fig. 5A). There were no significant differences between groups for the other metabolic categories “amino acid transport and metabolism” and “lipid transport and metabolism” (see Fig. 5B and 6C). The COG category “mobile: prophages, transposons” had significantly higher mean coverage for intestinal samples from sludge medium than from brewery medium, while the COG category “replication, recombination and repair” had significantly higher mean coverage for intestinal samples from sludge medium compared to the other substrates (Fig. 5D and 6E). The COG category “defense mechanisms” had no significant difference between substrates (Fig. 5F).

### Functional microbiome dynamics correlate with host survival and conversion

We investigated the dynamics of the metabolic capacity of microbiomes across sample types and substrates, revealing a lower metabolic capacity in the starting substrate, which reflects a smaller microbial community with less metabolic capacity (see Fig. 2A and 6A). Furthermore, we found similar levels of metabolic capacities between end substrate and intestinal samples, indicating a larger microbial community and higher metabolic capacity in both groups (see Fig. 2A and 6A). We correlated the metabolic capacity with host phenotypes, including survival and feed conversion, which revealed a positive correlation between the mean metabolic capacity of gut-related MAGs and both survival and feed conversion (see Fig. 6B). Interestingly, when correlating the mean metabolic capacity of all MAGs and both survival and feed conversion, we saw no significant correlation. This indicates that the phenotypes are specific to the gut-related MAGs (see Fig. S4).

We investigated the mean coverage of KEGG metabolic pathways (modules) related to gut-related MAGs, and their association with host survival and feed conversion (see Dats S8). We found several modules associated with host phenotypes, including “pyridoxal-P biosynthesis,” which had a positive correlation with both host survival and feed conversion (see Fig. 6C). Furthermore, our correlation analysis revealed a correlation between survival of the host and microbial drug resistance, more specifically “multidrug resistance, efflux pump Mexcb-OprJ,” and also aromatic amino acid metabolism, more specifically “tryptophan biosynthesis by chorismate” (see Fig. 6C). Subsequently, we found a negative correlation between the Entner-Doudoroff pathway, homoprotocatechuate degradation, and larval feed conversion (see Fig. 6C).

## DISCUSSION

The larval gut microbiome has been suggested to play a major role in insect production and bioconversion of organic waste and by-products (36, 37) as insects and their microbiomes have evolved together to convert a wide range of nutrient-poor diets into high-quality protein (11, 37). However, very little is known about how these insect-microbiome associations could be leveraged to optimize commercial insect production (38). We analyzed the microbiome composition and metabolic potential of house fly larvae grown on very different substrates (bran/deproteinized grass-based medium, a brewery by-product-based medium, and a digested sludge-based medium) using genome-resolved metagenomics, to understand the role of the microbiome in converting different waste and by-product-based substrates. Interestingly, the choice of growth substrate had a large effect on larval growth and metabolic performance traits (32). Substrate affected larval performance, performing better on brewer’s spent grain-based substrate regarding larval mass, larval yield, and substrate conversion efficiency. Wheat bran/deproteinized grass substrate did yield larger larvae than anaerobically digested sludge-based substrate, while anaerobically digested sludge-based substrate did yield higher net growth efficiency than wheat bran/deproteinized grass (32). This allowed us in the current study to relate changes in the larval gut microbiome with changes in larval performance.

One caveat of the analysis in the current study, though, is the low amount of data mapping to the MAG catalog; however, this limitation affects all samples equally, and a comparison to literature suggests that we caught the expected microbial diversity (discussed in section “Metagenomic studies need better MAG catalogs and higher power,” below). Therefore, we believe that it is reasonable to derive conclusions from the results.

### Larval gut enriched for stress-associated pathways of its microbiome

Gut-related MAGs were significantly enriched for the functions “multidrug resistance” and “phenylacetate degradation” with several other functional modules having higher coverage, though not significantly, including “trehalose biosynthesis,” “GABA biosynthesis,” “GABA shunt,” “glycogen biosynthesis,” “urea cycle,” and “glyoxylate cycle.” Multidrug resistance covers efflux pumps which can serve as protection against antimicrobial peptides (39, 40), the GABA shunt is known for protection against acids in prokaryotes (41), trehalose synthesis is known for stress protection in symbionts (42), and the glyoxylate cycle is important for bacteria in the viable but nonculturable state (43) indicating that gut-related bacteria do have mechanisms to cope with the strong microbial selection of the fly mid-midgut (44). This indicates the need for larval gut symbionts to contain pathways for coping with stress factors—an important consideration when selecting microbial strains for probiotics (29, 44).

### Substrate affects microbiome composition and metabolic potential

The effect of the substrate on microbial composition is well known in the waste-converting black soldier fly (45, 46) and has also been shown to affect the house fly microbiome (47). This is in accordance with observations that fly larvae microbiomes are highly dynamic with several transient microbes (48), and in agreement with our observations showing differences in MAG abundance and diversity between substrates. In addition, studies have shown that microbial function in black soldier fly larvae is also affected by substrate (49), in agreement with our observations of significant differences in metabolic potential between sludge-related gut samples and gut samples from other substrates. Interestingly, no major shifts in MAG metabolism were observed between substrates according to the UMAP projection of metabolic potential, concordant with the concept of different species being able to fulfill the same ecological niche (34). Conversely, substrate-specific differences in coverage of COG categories were observed, thus suggesting adaptation of the larval microbiomes to the different substrates.

The significantly higher mean coverage of “carbohydrate transport and metabolism” genes for gut samples of larvae reared on sludge medium when compared to brewery medium could be due to selection for microbes containing genes involved in synthesis and degradation of extracellular polymeric substances in the sludge-related samples (50). There were no significant differences in the coverage of this COG category between samples reared on brewery medium and bran/grass medium, nor bran/grass medium and sludge medium. This could be due to more complex carbohydrates in bran/grass medium than brewery medium, hence why the microbes need more genes for carbohydrate metabolism. Other COG categories of macronutrient metabolism (“lipid transport and metabolism” and “amino acid transport and metabolism”) had no significant differences in mean coverage, indicating that if substrate affects microbiome macronutrient metabolism, it is through carbohydrate content in the case of this experiment.

Gut samples of larvae reared on sludge medium additionally had significantly higher mean coverage of mobile elements (“mobile: prophages, transposons”) when compared to those reared on brewery medium, which could be a result of more bacteriophages being present in digested sludge (51). Mobile elements pose a risk of transferring antibiotic resistance genes (52), and therefore these results suggest the need for thorough analysis of the microbes associated with digested sludge if this substrate is applied for industrial rearing of fly larvae. Lastly, sludge-related samples also had a higher mean coverage of the COG category “replication, recombination, and repair.” The higher coverage may reflect a need for enhanced DNA repair mechanisms, potentially driven by the activity of mobile elements in sludge samples. This is unlikely to be due to increased replication, as there was no significant rise in the mean coverage of the COG category “cell cycle control, cell division, chromosome partitioning.” The KEGG module “multidrug resistance” was enriched in gut-related samples, which seems to be a general enrichment across all substrates, as the COG category “defense mechanisms” (which contains drug resistance genes) had no significant differences between substrates. Based on our observations, it could be hypothesized that adding non-native bacteria with the ability to degrade complex polymers to the sludge medium could bypass infection by the native phages or mobile elements and be able to degrade the polymeric substance, releasing more nutrients for fly larvae growth.

### Microbial metabolic potential correlates with larval phenotype

By reconstructing MAGs from the samples, it was possible to elucidate the functional role of the larval microbiome on larval growth and conversion of waste and by-products. The observed correlation between microbial pyridoxal-P synthesis (vitamin B_6_) and larval survival and conversion efficiency could indicate that this nutrient is a limiting factor for larval performance, as larval survival and substrate conversion efficiency increased with a higher coverage of this pathway. Vitamin synthesis is a well-known benefit of insect symbionts (11, 53). To verify the importance of pyridoxal-P synthesis, experiments with vitamin backfill could be performed in a similar manner as Pei et al. (24), who verified the positive effect of a strain of *Bacillus velezensis* on vitamin synthesis, by supplying germ-free black soldier fly larvae with riboflavin (vitamin B_2_). The negative correlation observed between coverage of the Entner-Doudoroff pathway and feed conversion could be an indication of an opportunistic fight for glucose, which might affect the feed conversion of the house fly larvae. Homoprotocatechuate is part of the metabolism of aromatic rings from metabolic products from aromatic amino acid degradation (40, 54), and might therefore cause a lack of essential aromatic amino acids for fly nutrition. In consequence, the larvae would need to ingest more feed to obtain the same amount of growth, thus causing the lower substrate conversion efficiency observed.

### Defining gut-related MAGs is challenging

Defining gut-related microbes is challenging in fly larvae, as these organisms live in their feed where they also excrete feces, making substrate and intestine samples similar in microbial composition. Consequently, gut-related microbes were defined based on the presence of substrate on day 0, of which there was a low sample size (one for each substrate), causing uncertainty in this definition. Knowing the true, resident gut microbiome from “passive passengers” helps elucidate which microbes thrive in the larval gut and might exert a beneficial function in the larval gut, potentially serving as targets for gut microbiome engineering (36, 55). This knowledge could be achieved by analyzing microbial dynamics, including the microbes present on eggs/neonates, fresh substrate, as well as a time series of larval guts and substrate samples (56). In addition, different gut segments could be analyzed to determine how different microbes react to the passage of the larval gut (57). Optimally, such an analysis would include a study on microbial activity to verify metabolic potential through transcriptomics, proteomics, or metabolomics.

### Metagenomic studies need better MAG catalogs and higher power

To our knowledge, only one study (58) has previously used genome-resolved metagenomics to investigate the importance of the larval gut microbiome of insects that have the potential for industrial waste conversion. By applying genome-resolved metagenomics, we were able to compare not only the taxonomic composition, but also the metabolic potential, of the microbiomes related to different substrates and larval guts. This was done to gain insight into how the microbiomes functionally adapt to the different conditions and additionally reveal how host performance is affected by microbiome functionality. This study, however, faced several limitations, one being the small sample size causing low statistical power. In addition, only 1.2% of the Illumina reads mapped to our MAG catalog meaning that lots of microbial information was not captured in this study, including the true diversity and functionality of the samples. This is likely an inherent limitation from our assembly strategy, where Nanopore reads originating from an independent growth experiment were used for the hybrid assembly. If there were significant differences in the microbiome between these two experiments, there would be bacterial species found in the experiment of this work lacking in the generated MAG catalog. All treatments were, however, similarly affected by this limitation, and therefore comparisons could be conducted on the information gained from the MAG catalog without introducing bias between samples. Comparing the taxonomy of the MAGs we were able to construct indicates that we captured most of the genera normally reported in house fly microbiome studies, including *Providencia* (found in seven studies), *Enterococcus* (found in four studies), *Morganella*, *Vagococcus*, *Microbacterium*, *Alcaligenes*, *Pseudomonas*, *Weissella*, *Ochrobactrum,* and *Sphingobacterium* (found in three studies) (47, 59–65). Genera commonly found in literature, but not in our MAG catalog include *Proteus* (found in five studies), *Staphylococcus* (found in four studies), *Serratia*, *Bacillus*, *Lactobacillus,* and *Corynebacterium* (found in three studies). This lack of genera could be due to an incomplete MAG catalog but could also be due to the source of microbes varying, as some studies use wild flies while others use laboratory flies, and within the literature, the fly larvae microbiome detected varies a lot, with only two genera (*Providencia* and *Proteus*) found in more than half of the studies. This suggests that we did capture most of the expected microbial diversity of the house fly larval gut. In addition to this, studies have also found that the presence of different bacteria is dependent on life stage (larvae, pupae, and adults) but also larval stage as well as origin of population and growth substrate (59, 66, 67). The importance of growth substrate on the bacterial composition of the larval gut is also evident in the present study, where, for example, *Coprothermobacter* and *Methanobacter* were mostly associated with the sludge-based substrate. These genera and genera such as *Pantoea*, *Cellulosimicrobium*, *Bordetella*, *Alcaligenes*, *Empedobacter*, *Leucobacter,* and *Sanguibacter* are also examples of novel genera not identified in traditional 16S rRNA studies (e.g., [59, 66, 67).

Optimally, an additional sequencing method like amplicon sequencing could be applied to catch the whole bacterial diversity, but this was not possible in the given study. It should also be noted that metabolic analyses on low-quality MAGs are not optimally compared to using high-quality MAGs (68), which we did not succeed in generating. We believe that increasing statistical power by increasing the sample size, generating a more representative MAG catalog, analyzing the different compartments of the larval gut, including multiple time series, and verifying results with activity data, like transcriptomics or proteomics, could unfold the true potential of genome-resolved metagenomics for revealing important microbes and microbial functions in reared fly larvae. By applying genome-resolved metagenomics for future studies on waste-converting fly larvae, we see a huge potential in revealing microbes with important functions in degradation of waste, fly health, and fly performance. By gaining insight into the underlying functionality of host-microbiome interactions in these species, it will also become possible to manipulate host-microbiome interactions, for example, by the addition of pro- or prebiotics, phage therapy, and selective breeding for an optimized microbiome (19). Improving waste-conversion outcomes through the use of microbiome-optimized fly larvae could have a huge impact on sustainability, effectively reducing waste and by-products and efficiently converting these into sustainable proteins suited for fish or livestock feed, paving the way for a greener future.

### Conclusion

We generated 154 MAGs from substrates and guts of house fly larvae reared on different waste and by-product-based substrates. MAGs associated with fly guts had higher coverage of functions related to stress responses, indicating a strong selection of microbes by the fly gut that should be considered when applying probiotics in the rearing of fly larvae. The microbiome of flies was affected by substrate, with the microbiome of larvae reared on sludge-based samples generally being more diverse in low-abundance species and having a higher metabolic capacity compared to other substrates. We also found significant differences in COG coverage between substrates, but clustering based on metabolic potential indicated that the microbial metabolism overall was similar between substrates, with different microbes fulfilling the metabolic functions between substrates. Lastly, we found both positive and negative correlations between the coverage of microbial pathways and larval phenotypes. Together, our results demonstrate the utility of metagenome-assembled genomes in analyzing the microbial communities of industrially reared fly gut microbiomes and show the potential for finding microbial function correlated with improved larval traits that can be selected for in industrial insect rearing, either through microbiome manipulations or by adding function-specific probiotics.

## MATERIALS AND METHODS

The microbiome of guts from house fly larvae reared on three different substrates for 6 days were sequenced (4–5 replicates per substrate consisting of 5 guts each) in addition to substrate samples before addition of eggs (one replicate per substrate) and after 6 days of larval growth (2–3 replicates per substrate) (see Fig. 1). The substrates used were a wheat bran/deproteinized grass-based medium, a brewery by-product-based medium, and a digested sludge-based medium. For further description of rearing of larvae, substrates, and larval performance across substrates, see reference (32).

### Rearing of house fly (*Musca domestica*)

House fly eggs were collected from a house fly laboratory culture at Aalborg University (19th generation, kept at 23°C, population size of approximately 1,000 flies each generation, fed bran/alfalfa medium [21.3% wheat bran, 10.7% ground alfalfa, 0.8% maltose, 0.5% yeast extract, and 66.7% water]) (origin of the culture described in reference 69). Eggs were collected over 6 h by adding a beaker with egg-collection medium (0.5% yeast extract, 0.8% maltose syrup, 10.7% ground alfalfa straw, 21.3% wheat bran, and 66.7% water) with a piece of cotton soaked in a solution of milk powder (15%) to the fly cages.

### Experimental setup

Larvae were grown in three different substrates (see Fig. 1). In short, plastic beakers (365 mL, *D* = 95 mm) were filled with 70 g of either of three growth substrates: Bran/grass medium (21.3% wheat bran, 10.7% deproteinized grass, 0.8% maltose, 0.5% yeast extract, and 66.7% water), brewery medium (8.3% wheat bran, 4.2% deproteinized grass, 0.3% maltose, 0.2% yeast extract, 67.0% brewer’s spent grain, and 20.0% water), or sludge medium (17.4% wheat bran, 8.6% deproteinized grass, 0.6% maltose, 0.4% yeast extract, 66% centrifuged sludge, and 7% water). Eggs were added to a density of 1 egg/g substrate, and the beakers were placed in a humidified Binder (KBWF 720) temperature cabinet. The light:dark cycle was set to 12:12 h, the temperature was 23.5°C ± 0.8°C (mean ± std), and the average humidity was 58.9% ± 5.3%. When a plateau in larval weight was measured, cultures were terminated by freezing.

### Survival and substrate conversion

Larval phenotypic data (weight, survival, and substrate conversion) were obtained from reference (32) and growth included in the current study (Data S1).

Survival was determined as the ratio of the total number of larvae and pupae in each replicate compared to the number of added eggs. As the larvae were frozen, it was assumed that any larvae that were alive when the cultures were terminated. In addition, larvae removed for gut extraction (described in “DNA sampling–growth substrate,” below) were included as survivors. Conversion was defined as the dry weight of larvae harvested per dry weight of growth substrate lost. As the larvae for gut extraction had been removed late in the experiment, the weight of larvae was defined as the weight of surviving larvae, in addition to the expected weight of the removed larvae had they gained the same mean weight as the surviving larvae.

### DNA sampling–growth substrate

After preparation of the three growth substrates, each was sampled into an Eppendorf tube (1.5 mL) and stored at −20°C. On day 6, substrate samples from three replicates for each of the three substrates were collected and stored in Eppendorf tubes (1.5 mL) at −20°C. Before DNA extraction, samples were thawed, and 100 mg was transferred with a sterilized spatula to a ZR BashingBead Lysis Tube (2.0 mm) (Zymo Research), and the samples were refrozen at −20°C.

### DNA sampling–insect guts

When larvae were 6 days old, five larvae were sampled per replicate for five replicates per growth substrate, and their guts were extracted. In short, this was done by surface sterilizing larvae for 30 s in 0.5% sodium hypochlorite and subsequently washing twice in sterile water for 30 s. The sterilization process was performed twice. The larvae were transferred to sterile narrow Drosophila vials (VWR) and anesthetized with a flow of CO_2_ into the tube. The guts were dissected by transferring the larvae to a sterile Petri dish, removing their head with a sterile scalpel, and pulling out the gut with sterile forceps. The guts were transferred to a ZR BashingBead Lysis Tube (2.0 mm) (Zymo Research) and stored at −20°C.

### DNA extraction, library preparation, and short-read sequencing

DNA was extracted from gut and substrate samples following the HostZERO Microbial DNA Kit protocol (Zymo Research), with some modifications. First, 750 µL DNA Elution Buffer (Zymo Research) was added to each ZR BashingBead Lysis Tube (2.0 mm), and the sample was bead-beaten for 60 s at 6.5 m/s on an MP Fastprep 24 (MP Biomedicals). The samples were spun down for 2 min at 16,000 RCF, and the supernatant was discarded. The pellet was resuspended in 100 µL DNA/RNA Shield (Zymo Research) and 750 µL ZymoBIOMICS Lysis Solution (Zymo Research), mixed, transferred to a ZR BashingBead Lysis Tube (0.1 and 0.5 mm), and bead beaten on the MP Fastprep for 5 min (5 rounds of 6.5 m/s for 60 s). The remaining extraction followed the HostZERO protocol. DNA concentrations were determined with a Qubit 4 fluorometer (Thermo Fisher Scientific) and DNA length with a TapeStation 4150 (Agilent). The DNA for Illumina sequencing was fragmented on a Covaris M220 with peak incident power: 30W, duty factor: 20%, cycles per burst: 50, duration: 120–150s. Fragment length was evaluated on a TapeStation 4150 (Agilent). Libraries were prepared on a MagicPrep NGS system (Tecan) using the Revelo DNA-Seq Mech for MagicPrep NGS kit (Tecan) following the manufacturer’s protocol. Samples were sequenced by Novogene (Illumina NovaSeq PE 150), aiming for at least 10 Gb per sample.

### Nanopore cultures, library preparation, and long-read sequencing

To gain enough DNA for long-read nanopore sequencing, additional larval cultures were grown independent of the ones used for collecting phenotypic and Illumina sequencing data. Plastic beakers (365 mL, *D* = 95 mm) were filled with 70 g of either of three growth substrates bran/grass medium (21.3% wheat bran, 10.7% deproteinized grass, 0.8% maltose, 0.5% yeast extract, and 66.7% water), brewery medium (12.8% wheat bran, 6.4% deproteinized grass, 0.5% maltose, 0.3% yeast extract, 67.0% brewer’s spent grain, and 13.0% water), or sludge medium (14.1% wheat bran, 7.0% deproteinized grass, 0.5% maltose, 0.4% yeast extract, 67% centrifuged sludge, and 11% water). Eggs were added to a density of 1 egg/g substrate, and the beakers were placed in a P selecta incubator (Buch & Holm) with water-containing trays at the bottom. The light: dark cycle was set to 12:12 h light dark, the average temperature was 24.1°C ± 0.9°C, and the average humidity was 81.4 ± 7.1%.

Gut samples were sampled on days 7 and 8 using the same procedure as described earlier. DNA was extracted with a HostZERO Microbial DNA Kit (Zymo Research) with previously described modifications. DNA samples from replicates reared on the same substrate were pooled to increase DNA mass. The pooled sample for sludge samples had a high abundance of short fragments and was purified with size exclusion using AMPure XP beads (Beckman Coulter) at a 0.4× ratio with L Fragment Buffer (Oxford Nanopore Technologies) for elution. The size exclusion was performed twice. Libraries for Nanopore sequencing were prepared using a Native Barcoding Kit 24 V14 (Oxford Nanopore) following the manufacturer’s protocol. The samples were sequenced with a PromethION Flow Cell (R10.4.1, FLO-PROM114M) on a PromethION P24-A100 machine running Minknow v. 22.12.5 at 400 bp/s with a 4 kHz sample rate. Base calling was done with Guppy v. 6.4.6 with the super accuracy model.

### Bioinformatics

#### Quality control

Raw sequence Illumina reads were quality controlled, using FastQC (v0.11.8, http://www.bioinformatics.babraham.ac.uk/projects/fastqc/) to assess filtering and quality steps. Adapters and low-quality reads were removed with Trimmomatic (70), with a quality base of 30 and a minimum length of 50 bases. We removed duplicates, and reads were re-paired to remove singletons using bbmap v.38.35 (https://sourceforge.net/projects/bbmap/). We filtered data for the eukaryotic data, using a k-mer approach with Kraken2 v2.1.2 (71), to increase assembly efficiency by reducing eukaryotic contaminants.

#### Genome-resolved metagenomics

A hybrid genome assembly approach was implemented to combine the high accuracy of Illumina short reads with the long-read sequencing capabilities of Oxford Nanopore Technologies. This workflow involves quality control, error correction, *de novo* assembly, polishing, and final hybrid assembly merging to produce high-quality genome assemblies. Oxford Nanopore long reads were assembled using Flye (72), an assembler optimized for error-prone long reads. The assembly was run with the --meta flag to account for metagenomic data. To improve the accuracy of the long-read assembly, Racon (73) was used for consensus sequence polishing. Before polishing, long reads were aligned back to the Flye assembly using Minimap2 (74). To leverage the accuracy of Illumina short reads, the long-read assembly polished with Racon was used as input for SPAdes hybrid assembly (75). Short reads were incorporated to improve contiguity and base accuracy. The SPAdes hybrid assembly was merged with the long-read Flye assembly using Quickmerge (76), a tool designed to combine assemblies while preserving the best structural information.

Assembled contigs were quality assessed with Quast (77). Filtering for a minimal length of 1,000 bases per scaffold was applied. To increase effective metagenomics binning, we used the anvi’o pipeline (78, 79) with automatic binning, using Metabat2 (80), followed by manual curation with the anvi’o platform. Briefly, (i) anvi’o was used to identify genes in the scaffolds using Prodigal v2.6.3 (81) with default parameters. Subsequently, HMMER v3.3 (82) was used to identify genes matching archaeal, protists, and bacterial (83) single-copy core gene (SCGs) collections with hidden Markov models (HMMs). Also, ribosomal RNA-based HMMs were identified using barrnap (https://github.com/tseemann/barrnap). The HMMs of SCGs were used to determine the completeness and redundancy of MAGs; (ii) read recruitment of the metagenome to the scaffolds was carried out using BWA v0.7.1596 (84) (minimum identity of 95%) and samtools (85). We binned contigs automatically using Metabat2 (80). Each Metabat2 bin was manually curated using the anvi’o interactive interface to ensure high completion and low redundancy. The interface considers each scaffold’s sequence composition, differential coverage, GC content, and taxonomic signal (78, 86). We defined all bins with >50% completeness as MAGs. We further classified the MAGs according to the MIMAG criteria (35). Ribosomal RNAs were already annotated, and tRNAs were annotated using tRNAscan-SE v2.0.12 (87).

#### Identification, refinement, taxonomic, and functional inference of MAGs

We used anvi’o to infer the taxonomy of MAGs based on the proximity of single-copy gene markers based on the Genome Taxonomy Database (GTDB) (88). Subsequently, we applied Kaiju (89) with NCBI’s non-redundant protein database “nr” to infer the taxonomy of genes (as described in http://merenlab.org/2016/06/18/importing-taxonomy/). For functional inference, we used clusters of orthologous (COGs) (90), the Kyoto Encyclopedia of Genes and Genomes (KEGG) (91), and protein families (Pfam) (92), which were annotated through the anvi’o platform. A summary of the MAGs generated for this study is available at Dats S4.

#### MAG phylogenetic relationships

Phylogenetic analysis was based on the nucleotide sequences of the single-copy gene *Adenylsucc_synt* (PF00709.21), which was exported from anvio’s and aligned for all MAGs with MACSE v2.0.7’s (93) function *alignSequences* with default settings. Alignments were used to generate phylogenetic trees with IQ-tree v3.0.1 (94) with ModelFinder (95) for determining the substitution model and 1,000 rounds of bootstrapping for determining branch support. The phylogenetic tree was visualized in Fig-Tree v1.4.4 and is available in Fig. S1.

#### MAG quality overview

MAG quality was assessed using metrics such as N50, total length, number of contigs, and completion percentage. Histograms for these metrics were generated using log-scaled data for better visualization (see Fig. S5). The abundance of MAGs was based on the mean coverage across each MAG. Data were normalized based on MAG length and were sum-normalized before analysis based on TMP normalization, using the r-package ADImpute (96). Rarefaction curves were estimated using the *r-package vegan* (97) to infer suitable sequencing depth and can be found in Fig. S2.

#### PCoA analysis for MAGs

A PCoA was performed to explore compositional differences in the microbiome across samples. A binary distance matrix was used, and the samples were visualized with colors corresponding to the substrate. Data were scaled and centered. An additional PCoA with a Euclidean distance matrix was generated and can be found in Fig. S4.

#### Hill’s diversity analysis

Hill’s diversity indices (q0, q1, and q2) were calculated for each sample to assess the diversity of the microbial communities. Boxplots were generated to compare the diversity indices across different sample types. Additionally, for a subset of samples from larval intestines, diversity was further analyzed based on growth substrate. Diversity indices were computed using the hill_div function (98), and the results were visualized using boxplots with jitter to show individual sample values.

#### Heatmap of microbial genera

A heatmap was generated based on the summed normalized abundance of each genus present in the MAG catalog. Information on sample type and substrate was added with the R package ComplexHeatmaps (99).

#### Prevalence analysis

The fly gut-related MAGs were identified by comparing the prevalence of MAGs across different sample types. Prevalence was defined as the proportion of samples in which a MAG or genus was present above a threshold of 0.0001 in terms of relative abundance.

For the analysis, MAGs with a prevalence greater than 75% in fly gut samples and not present in the start substrate samples were identified as gut-related. The classification of gut-related MAGs was subsequently used for enrichment analyses.

#### Differential abundance testing across substrates

Pairwise differential abundance testing was performed on MAGs across different substrates (brewery, bran/grass, and sludge) using the Wilcoxon rank-sum test. This non-parametric test was chosen because it does not assume normality in the data, which is appropriate given the typically skewed distribution of microbial abundance data. Pairwise comparisons were made between the three substrate groups, and *P*-values were adjusted using the False Discovery Rate (FDR) method. MAGs with significant differences (*P*.adj < 0.1) were visualized with those below the 0.05 threshold highlighted as statistically significant. The Wilcoxon rank-sum test was used to account for small sample sizes and potential deviations from a normal distribution. All analyses were conducted in R using rstatix for statistical testing and ggplot2 for visualization (100, 101).

#### Metabolic completion index for MAGs and gut-related MAGs

The metabolic completion index (MCI) was calculated to assess the metabolic potential of MAGs in fly gut samples. For each MAG, pathway completeness was derived from KEGG modules, averaged, and normalized by genome completion. MAG presence/absence was used to compute the summed metabolic capacity for each sample. The analysis was conducted for all MAGs and separately for gut-related MAGs.

#### UMAP analysis of MAGs

UMAP was applied to project MAGs into a 2D space to assess metabolic capacities across samples. To explore substrate-related differences, MAGs were colored by fold change between substrates (brewery, bran/grass, and sludge). Fold change was calculated based on MAG abundance across the different substrate conditions. Based on the Wilcoxon rank-sum test, non-significant differences were colored in gray.

#### Enrichment analysis

The statistical approach for enrichment analysis is previously defined (102). Briefly, the program anvi-compute-functional-enrichment determined enrichment scores for KOfams of low- and high-abundance by fitting a binomial generalized linear model (GLM) to the occurrence of each KOfam in each group and then computing a Rao test statistic. Functional enrichment analysis was performed on KEGG modules associated with gut-related and non-gut-related MAGs. Enrichment scores were calculated, and modules with an enrichment score >2 and adjusted *q*-value < 0.05 were considered significant. Volcano plots were generated to visualize the enrichment scores against −log(*q*-values).

#### Functional data aggregation and normalization

Gene coverage data were loaded from MAGs using gene caller IDs, and sequencing depth normalization was applied. Functions were annotated using the COG (Clusters of Orthologous Groups) database, focusing on COG functions, categories, and pathways. The data were processed and normalized by sequencing depth using total read counts per sample.

The normalized gene coverage was summarized by COG functional annotations, and the results were grouped to provide a comprehensive overview of gene functions across samples. COG functions, categories, and pathways were further merged to extract distinct functional profiles of the microbial community.

#### Differential COG analysis across substrates

The differential abundance of COG functions across substrates (brewery, bran/grass, and sludge) was analyzed using pairwise t-tests, with comparisons made between brewery vs. bran/grass, bran/grass vs. sludge, and sludge vs. brewery. *P*-values were adjusted using the Benjamini-Hochberg method, and fold changes were calculated as the ratio of COG coverage between substrate groups. Significant COG functions were defined as those with an adjusted *P*-value < 0.05 and a log2 fold change > 1 or < −1. The results were visualized using volcano plots for each comparison, displaying log2 fold changes against the -log of adjusted *P*-values. A *P*-value histogram provided an overview of significance distribution, and counts of significant COG functions were summarized by direction of change. The analysis was conducted using dplyr and reshape2 for data manipulation, DESeq2 for statistics, and ggplot2 and cowplot for visualization (101, 103–105).

#### Comparison of COG category coverage between substrates

The coverage of COG categories from intestinal samples was summed for samples from larvae reared on the same substrate. The summed coverages were subsequently compared between substrates using a Dunn test and visualized as boxplots with added *P*-values. In addition, mean coverage of the COG categories in start- and end-substrate samples was added to the plots as a reference.

#### KEGG module correlation with survival and conversion

To assess the relationship between KEGG module coverage and survival or conversion rates in fly gut samples, Pearson correlation was applied. For each KEGG module, linear regression was performed, and Pearson’s correlation coefficients were calculated.

Mean KEGG module coverage was plotted against survival rates, with regression lines and confidence intervals included to evaluate associations. Horizontal dashed lines represented the baseline mean coverage in the starting and end substrates for comparison. A similar method was applied to conversion rates, with KEGG module coverage plotted against conversion, regression lines fitted, and correlation coefficients calculated. Baseline coverage from start and end substrate was again included for context.

#### Correlation of MCI with survival and conversion

The relationship between the MCI and key phenotypes (survival and conversion) in fly gut samples was analyzed using Pearson correlation and linear regression. Summed metabolic capacity was plotted against survival and conversion rates for both overall MAGs and gut-related MAGs. Regression lines and confidence intervals were fitted to assess the strength of these relationships, with Pearson correlation coefficients and regression equations displayed on the plots.

#### Statistical assumptions

Statistical assumptions for parametric and non-parametric analyses were tested using Levene’s test for homogeneity and the Shapiro-Wilk test for normality of data.

### Highlights

- Genome-resolved metagenomic analysis of house fly larva gut microbiome
- Gut microbiome metabolic potential indicates strong selection
- Substrate affects the metabolic potential of the larval gut microbiome
- Larval survival and conversion correlate with microbial vitamin B_6_ genes

## Data Availability

Metagenome-assembled metagenomes are available at Zenodo with accession numbers 10.5281/zenodo.18877919 and 10.5281/zenodo.18878344.

## SUPPLEMENTAL MATERIAL

Figure S1 aem.00011-26-s0001.pdf Phylogenetic relationship between constructed MAGs.

Figures S2 to S5 aem.00011-26-s0002.docx Rarefaction curves of each sequenced sample, principal coordinate analysis of metagenomes, correlation between metabolic capacity and feed conversion and survival of larvae, and quality metric of assembled MAGs.

Supplemental data sets aem.00011-26-s0003.xlsx Data S1 to S8.

Supplemental legends aem.00011-26-s0004.docx Descriptive legends for Fig. S1 to S5.

ASM does not own the copyrights to Supplemental Material that may be linked to, or accessed through, an article. The authors have granted ASM a non-exclusive, world-wide license to publish the Supplemental Material files. Please contact the corresponding author directly for reuse.
