# Supplementary figures and images for: Functional gut microbiomes enhance performance in house fly larvae
Source: Appl Environ Microbiol. 2026 Apr 13;92(5):e00011-26. doi: 10.1128/aem.00011-26 (PMC13188918; doi:10.1128/aem.00011-26)

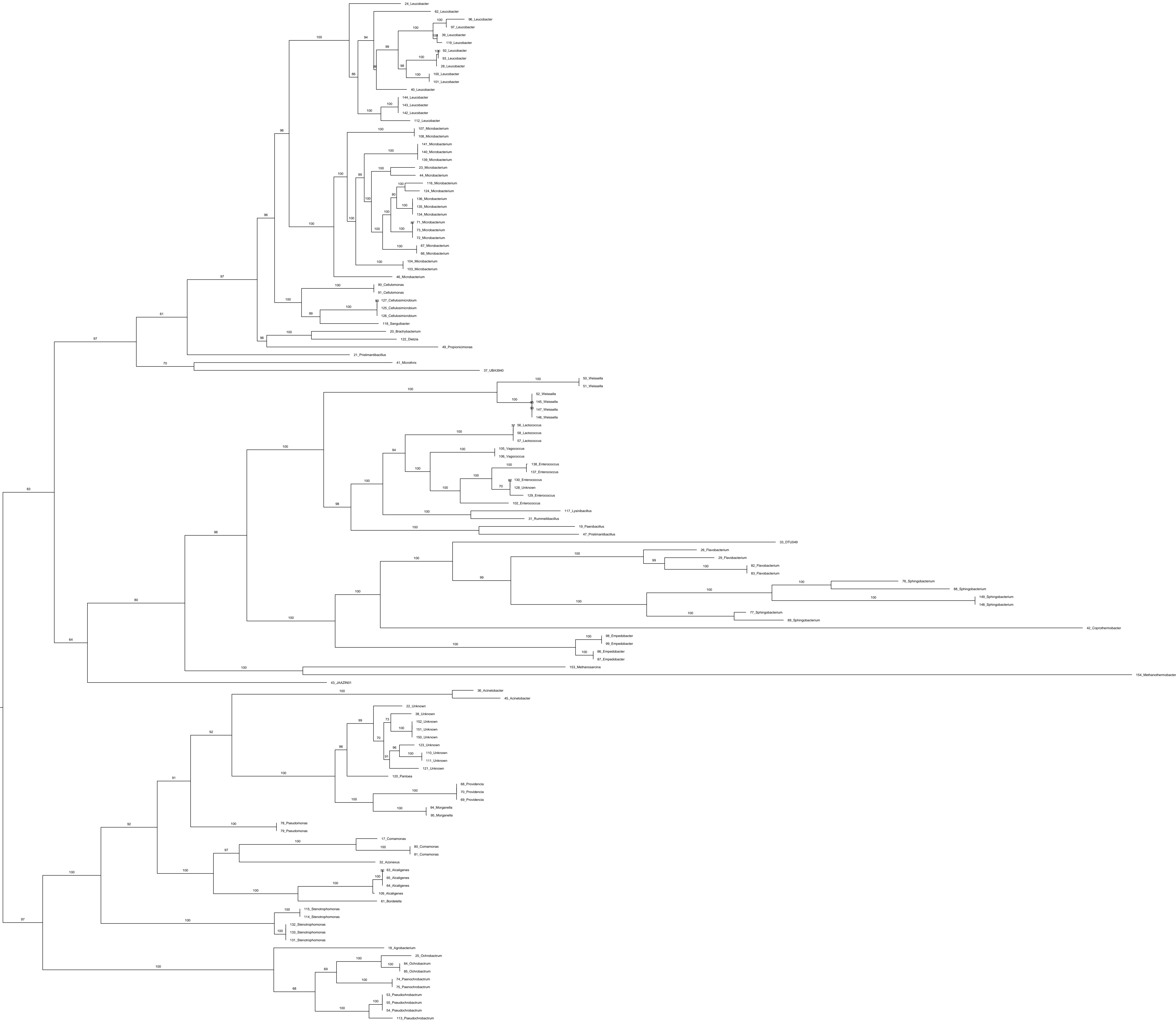

Supplement: Figure S1 — Phylogenetic relationship between constructed MAGs. [file aem.00011-26-s0001.pdf]

Figure S2


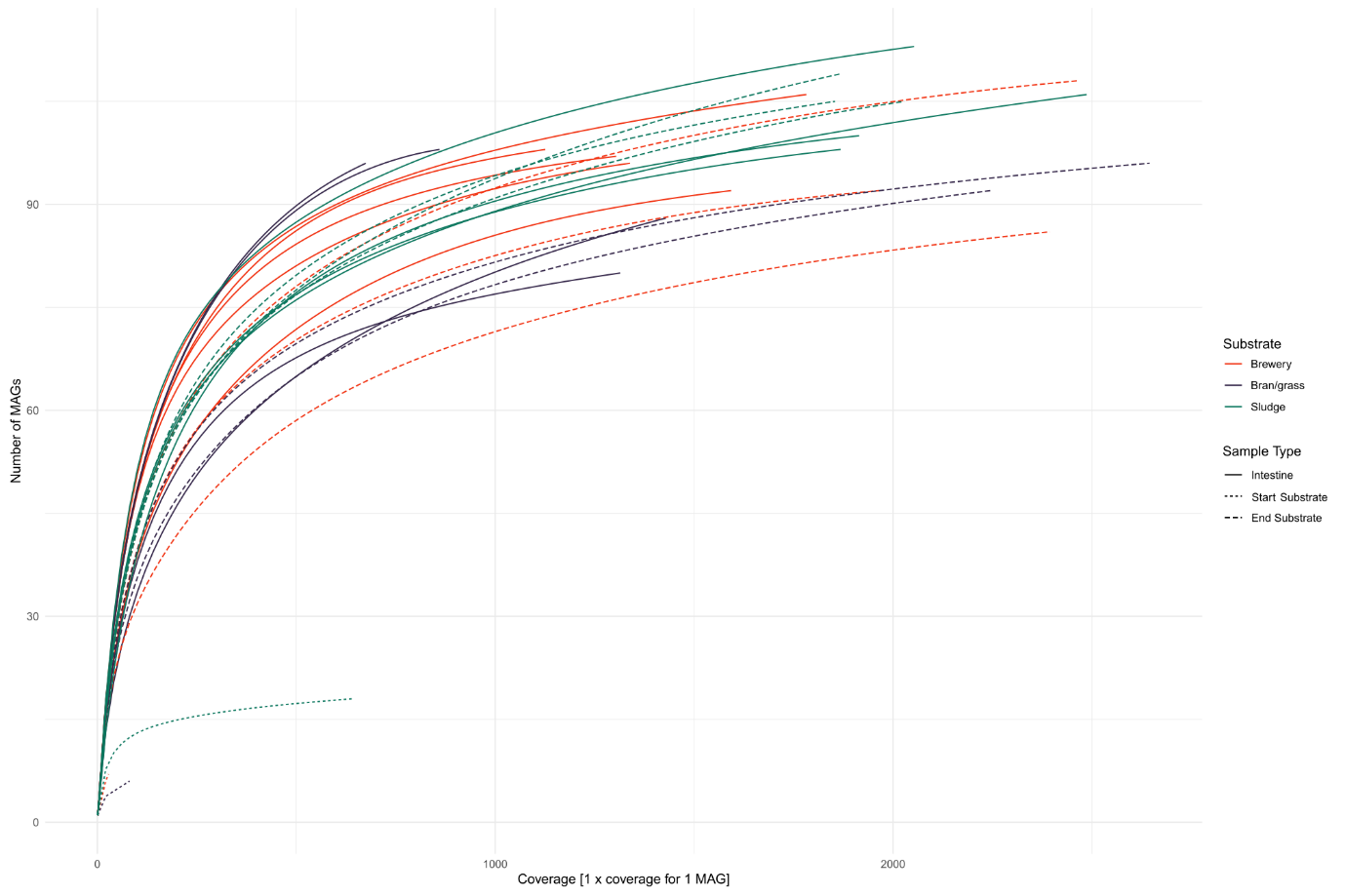


Figure S3


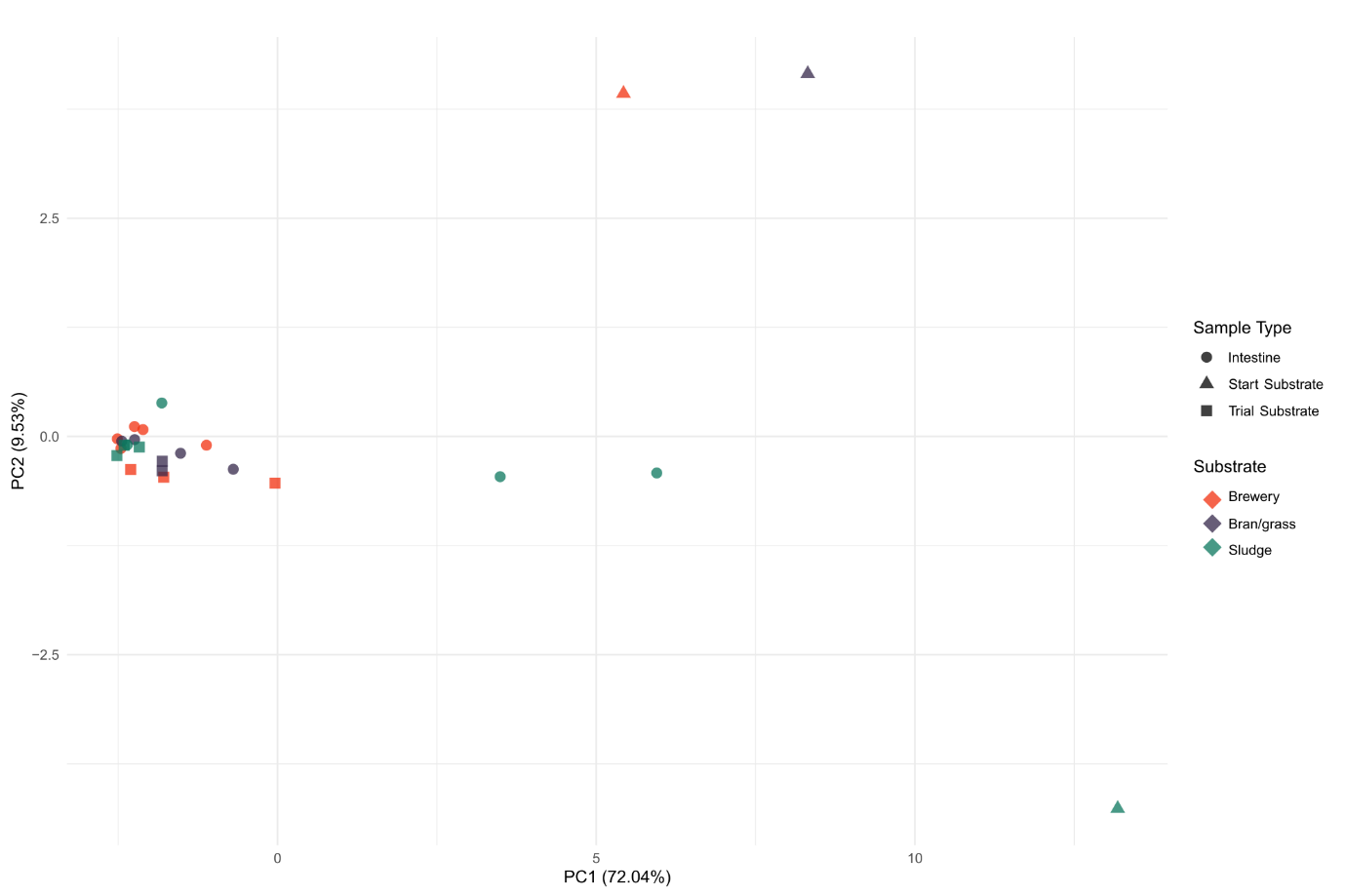


Figure S4


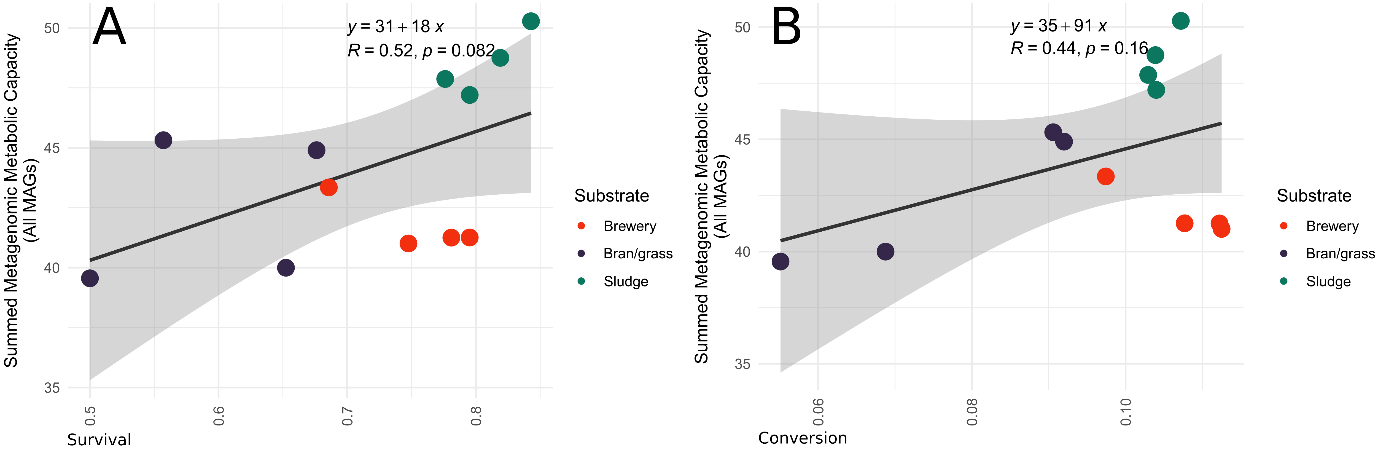


Figure S5


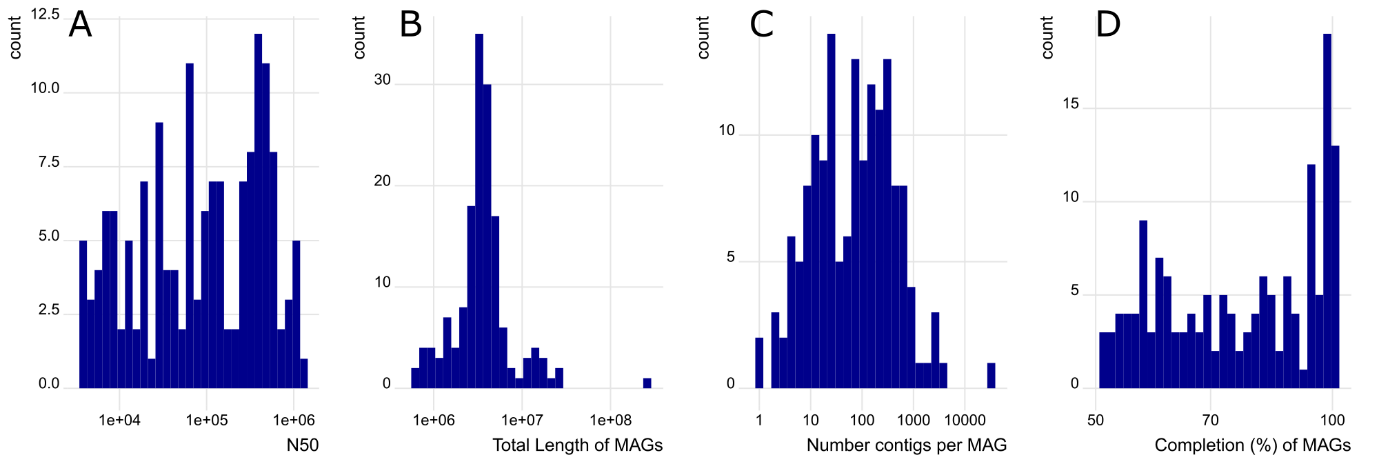

Supplement: Figures S2 to S5 — Rarefaction curves of each sequenced sample, principal coordinate analysis of metagenomes, correlation between metabolic capacity and feed conversion and survival of larvae, and quality metric of assembled MAGs. [file aem.00011-26-s0002.docx]
