# Supplementary material for: Functional gut microbiomes enhance performance in house fly larvae
Source: Appl Environ Microbiol. 2026 Apr 13;92(5):e00011-26. doi: 10.1128/aem.00011-26 (PMC13188918; doi:10.1128/aem.00011-26)
Supplement: Supplemental legends — Descriptive legends for Fig. S1 to S5. [file aem.00011-26-s0004.docx]

***Supplementary Figure S1 Phylogenetic relationship between constructed MAGs.*** *The phylogenetic distance between MAGs was determined based on alignments of the nucleotide sequence of the bacterial HMM gene for Adenylosuccinate Synthase. Values indicate branch support.*

***Supplementary Figure S2 Rarefaction curves of each sequenced sample.*** *Each curve designates the number of MAGs likely to be retrieved from a sequencing depth equal to the coverage of an average MAG. The line type indicates the different sample types and colouring indicates the different substrates.*

***Supplementary Figure S3 Principal coordinate analysis of metagenomes.*** *Principal Coordinate Analysis (PCoA) of metagenomes, based on the abundance of MAGs, related to sample types and substrate. The shape indicates the different sample types and colouring indicates the different substrates.*

***Supplementary Figure S4 Correlation between metabolic capacity and feed conversion and survival of larvae.*** *Scatterplot of mean metabolic capacity for each sample of all substrates correlated with A) survival and B) conversion of feed. The diagonal lines indicate a linear regression. The pale grey area indicates the confidence intervals of the regression.*

***Supplementary Figure S5 Quality metric of assembled MAGs.*** *Histogram of A) the N50 values of MAGs, B) the length of MAGs, C) the of number of contigs per MAG, D) the percentage completion of MAGs based on single copy core genes for the generated MAG-catalogue.*
